# Effects of Salinity and Abscisic Acid on Lipid Transfer Protein Accumulation, Suberin Deposition and Hydraulic Conductance in Pea Roots

**DOI:** 10.3390/membranes11100762

**Published:** 2021-10-01

**Authors:** Guzel R. Akhiyarova, Ruslan S. Ivanov, Igor I. Ivanov, Ekaterina I. Finkina, Daria N. Melnikova, Ivan V. Bogdanov, Tatyana Nuzhnaya, Tatiana V. Ovchinnikova, Dmitriy S. Veselov, Guzel R. Kudoyarova

**Affiliations:** 1 Ufa Federal Research Centre, Ufa Institute of Biology, Russian Academy of Sciences 450054, pr. Octyabrya, 69, 450054 Ufa, Russia; akhiyarova@rambler.ru (G.R.A.); ivanovirs@mail.ru (R.S.I.); i_ivanov@anrb.ru (I.I.I.); tanyawww89@mail.ru (T.N.); veselov@anrb.ru (D.S.V.); 2 M. M. Shemyakin & Yu. A. Ovchinnikov Institute of Bioorganic Chemistry, Russian Academy of Sciences, Miklukho-Maklaya str., 16/10, 117997 Moscow, Russia; finkina@mail.ru (E.I.F.); d_n_m_@mail.ru (D.N.M.); contraton@mail.ru (I.V.B.); ovch@ibch.ru (T.V.O.); 3 Ufa Federal Research Centre, Institute of Biochemistry and Genetics, Russian Academy of Sciences, Prospekt Oktyabrya 71, 450054 Ufa, Russia

**Keywords:** garden pea, *Pisum sativum*, lipid transfer proteins (LTPs), plant roots, plant hormones, abscisic acid (ABA), suberin, salt stress

## Abstract

Lipid transfer proteins (LTPs) participate in many important physiological processes in plants, including adaptation to stressors, e.g., salinity. Here we address the mechanism of this protective action of LTPs by studying the interaction between LTPs and abscisic acid (ABA, a “stress” hormone) and their mutual participation in suberin deposition in root endodermis of salt-stressed pea plants. Using immunohistochemistry we show for the first time NaCl induced accumulation of LTPs and ABA in the cell walls of phloem paralleled by suberin deposition in the endoderm region of pea roots. Unlike LTPs which were found localized around phloem cells, ABA was also present within phloem cells. In addition, ABA treatment resulted in both LTP and ABA accumulation in phloem cells and promoted root suberization. These results suggested the importance of NaCl-induced accumulation of ABA in increasing the abundance of LTPs and of suberin. Using molecular modeling and fluorescence spectroscopy we confirmed the ability of different plant LTPs, including pea Ps-LTP1, to bind ABA. We therefore hypothesize an involvement of plant LTPs in ABA transport (unloading from phloem) as part of the salinity adaptation mechanism.

## 1. Introduction

Lipid transfer proteins (LTPs) belong to a family of small, ubiquitous proteins that reversibly bind phospholipids and fatty acids within their hydrophobic cavity [1]. LTPs’ capacity for transfer of lipids between membranes in vitro suggests they participate in lipid trafficking both within the cell and also in the apoplast, intercellular space and plant phloem [2,3]. However, the precise biological role of LTPs in vivo is still unclear. In plants, preferential LTP localization on the outside of the plasmalemma [4] suggests their involvement in transfer of monomers required for the assembly of water impermeable lipid barriers such as suberin lamellae deposited in the root endodermis. However, involvement of LTPs in suberin deposition remains speculative and requires further investigation.

Apoplastic barriers such as suberin lamellae protect against uncontrolled flow of water and solutes under abiotic stresses [1]. Responsiveness of LTP genes to drought and salinity stress [5,6] implies an involvement in stress adaptation. Nevertheless, importance of LTPs for suberin deposition under drought is mentioned only rarely [1,7], whilst no attempt seems to have been made to relate LTP-dependent formation of apoplast barriers to salt stress responses even though enhanced deposition of suberin and lignin in apoplastic root barriers is an important component of salt tolerance [8]. Transgenic tobacco overexpressing an LTP gene has been shown to accumulate low Na^+^ level upon exposure to high salinity [9]. However, in this report deposition of apoplast barriers was not considered as possible mechanism for excluding the sodium.

A further gap in our understanding of the involvement of LTP-dependent suberin deposition in salt resistance concerns its effect on root hydraulic conductance. Overexpression of LTP gene has been shown to enhance drought tolerance of *Nicotiana tabacum* by slowing transpiration [9]. Decreased transpiration improves salt tolerance by reducing the delivery of toxic ions delivered to the shoot system [10]. One cause of the slow transpiration can be a decrease in root hydraulic conductance. This is known to depend on deposition of apoplast barriers and to influence transpiration rate [11].

The hormone abscisic acid (ABA) is important for successful adaptation to abiotic stresses, including salinity [12]; its action being realized through the expression of protective proteins, one of which is considered to be LTPs. ABA has been shown to induce expression of LTP genes in *Tamarix hispida* [13]. *Arabidopsis thaliana* plants overexpressing LTP3 have elevated level of endogenous ABA [2]. We reported that salt treatment simultaneously increased both LTPs and ABA abundance accompanied by increased deposition of suberin in roots of pea plants [14]. These results suggest an involvement of ABA in the induction of either LTP accumulation or suberin deposition as well as a possible participation of LTPs in promoting ABA accumulation. It is known that LTPs are able to bind hydrophobic signaling molecules such as jasmonates and may take part in signal transduction in plants by aiding jasmonates transport through vasculature [15]. However, the capacity of LTPs to bind to such a hydrophobic compound as ABA has not been studied yet.

To clarify these relationships we followed the effects of salt treatment on LTPs and ABA localization and abundance in pea roots in relation to suberin deposition and hydraulic conductance of root. To test whether accumulation of ABA is involved in the control of LTPs abundance in salt-stressed plants we compared these outcomes to the effects of ABA treatment. This research also tests the possibility that ABA is involved in the control of LTP-mediated root suberization and that LTPs influence ABA trafficking. Accordingly, we investigated the binding of ABA with various LTPs from different plant species.

## 2. Materials and Methods

### 2.1. Materials

ABA and TNS (2-p-toluidinylnaphthalene-6-sulphonate) were purchased from Sigma-Aldrich (St. Louis, MO, USA). The recombinant Ps-LTP1, Ag-LTP and Lc-LTP2 were overexpressed in *Escherichia coli* and purified as described previously [3,16,17].

### 2.2. Plant Growth Conditions and Treatments

Seeds of the garden pea *Pisum sativum* (cultivar “Sacharniy 2” supplied by “Udachnye semena", Novokujbyshevsk, Russia) were soaked in water for 24 h, then wrapped with wet gauze to germinate. Four days after the start of soaking, pea seedlings were transplanted on to rafts (disks made of foam polyethylene with holes for plants), placed in trays with tap water under a 14-h photoperiod of 400–500 μmol m^−2^ s^−1^ PAR (ZN-500 and DNAT-400 lamps) at 24/18 °C (day/night). After seven days, rafts with seedlings were transferred to 500 mL beakers with 10% Hoagland-Arnon solution. Part of beakers contained 50 mM sodium chloride or 100 µM ABA. Solutions were changed daily. At the time of transfer, no suberin had yet been deposited in the roots.

### 2.3. Characteristics of Water Relations

To measure transpiration, beakers with 8–9 seedlings (10 beakers for each variant of treatment) were covered with polyethylene film with holes for plants to prevent evaporation from water surface and transpiration was assessed gravimetrically as weigh loss by the beakers over 2 h. Transpiration was measured during five days and when it was decreased by either salt or ABA treatment (which happened on the fourth and the second day after the start of the treatments, respectively) hydraulic conductance was measured and roots were sampled for suberin staining, immunolocalization and PCR analysis.

Water potential of disks cut from the second differentiated leaf counting from the bottom was measured with a psychrometer (PSYPRO, Wescor, USA). The hydraulic conductance of the water transport pathway from roots to leaves was calculated, as described [18], using the formula: L = T/[(Ψs − Ψl)], where T is transpiration, and Ψs and Ψl are the water potential of the nutrient solution and leaf, respectively. Significant differences between means were analyzed by a t-test.

### 2.4. Microscopic Study

For immunolocalization of LTPs and ABA pieces cut from basal part of roots were fixed in 0,1 M phosphate buffer (PB) pH 7.2 containing 4% N-(3-dimethylaminopropyl)-N’-ethylcarbodiimide hydrochloride (Merck, Darmstadt, Germany) for 12 h at 4 °C and then in 4% paraformaldehyde (Riedel de Haen, Seelze, Germany) and 0.1% glutaraldehyde (Sigma, Steinheim, Germany). Fixed root tissues were then washed three times with phosphate buffer and after dehydration in series of increasing ethanols were embedded in JB4 resin (Electron Microscopy Sciences, Hatfield, PA, USA). Histological sections 1.5 μm thick were obtained using a rotary microtome (HM 325, MICROM Laborgerate, Germany). After applying blocking solution for 30 min (phosphate buffer containing 0.2% gelatin and 0.05% Tween-20) root sections were incubated with polyclonal rabbit anti-LTP (1:200 dilution) [16,19] or anti-ABA [20] (1:80 dilution) sera overnight at 4 °C. The interaction of polyclonal rabbit anti-LTP antiserum with recombinant protein was evaluated previously by means of Western blotting and ELISA assays [16,19]. Specificity of immunostaining for ABA was confirmed previously by increased staining in plant treated with exogenous ABA (positive control) as well as by decreased staining in the case of ABA-deficient mutant [20]. Slides were then washed three times in 0.1 M phosphate buffer with 0.05% Tween-20 followed by 3 h incubation at 37 °C with anti-rabbit IgG secondary antibodies conjugated to Alexa Fluor 555 (Invitrogen, Rockford, USA) The slices were then rinsed again five times with PB, covered with glass and then imaged by confocal microscopy using an FV3000 Fluoview (FV31-HSD) (Olympus, Tokyo, Japan) and laser excitation line of 561 nm. Fluorescence emission was detected at 568 nm. Detection was in integration frame mode for imaging with a count of 8. To facilitate discrimination between LTPs and ABA detected by the same label (Alexa 555), we assigned them to different pseudocolors: LTPs to magenta and ABA to cyan.

The average fluorescence intensity of the images taken with the confocal microscope in phloem region was estimated using the ImageJ software (National Institutes of Health, Bethesda, MD, USA, https://imagej.nih.gov/ij, accessed on 20 September 2021). The fluorescence was expressed in arbitrary units, with maximal staining fluorescence taken as 100 % and minimal as 0. Suberin in the free–hand root cross-sections was stained dark orange with alcoholic Sudan III (Sigma, USA) [21]. 

### 2.5. RNA Extraction and Analysis of Abundance of LTP mRNA

RNA was extracted from roots on the second and fourth day after the start of ABA/salt treatments respectively, using TRIzol™ Reagent (Sigma) according to the manufacturer’s instructions. Potential contaminating DNA was digested with DNaseI (Synthol, Moscow, Russia) and airst-strand cDNA was synthesized using the M−MLV reverse transcriptase (Fermentas, USA). Oligo(dT)15 was used as a primer, and the reverse transcription reagents were incubated at 37 °C for 1 h in a total volume of 25 μL. After ten-fold dilution, 2 μL of the synthesized cDNA was used for quantitative real-time polymerase chain reaction (qPCR). The primers for qPCR were designed based on the cDNA sequence [19] using the PrimerQuest™ Tool. The following primers were used for quantitative analysis of Ps-LTP1 (GenBank accession number KJ569141) pea gene expression: forward 5’- GCTGCCGGTTCTATTCCTAAA-3’ and reverse 5’- GTTGGTGGAGGTACTGATCTTG -3. Quantitative PCR was performed by polymerase chain reaction in real time using a set of predefined reagents EvaGreenI (Synthol, Russia) and a CFX Connect real-time PCR Detection System (BioRad Laboratories, USA). The qPCR program was as follows: 95 °C for 5 min; 40 cycles of 95 °C for 15 s and at 60 °C for 20 s and 72 °C 30 s. After the final PCR cycle, a melting curve analysis was conducted to determine the specificity of the reaction (at 95 °C for 15 s, 60 °C for 1 min and 95 °C for 15 s). The efficiency of each primer pair was determined using 10-fold cDNA dilution series to reliably determine the fold changes. The β-tubulin gene (GenBank accession number: X54844) was chosen as an internal control to normalize the amount of total RNA present in each reaction (primers: forward—GCTCCCAGCAGTACAGGACTCT; reverse—TGGCATCCCACATTTGTTGA). The quantification of gene expression was performed using CFX Connect real-time PCR Detection System (BioRad Laboratories). All reactions, including the non-template control, were performed three times. The threshold values (CT), generated from the CFX Connect real-time PCR Detection System software tool (Applied Biosystems) were employed to quantify relative gene expression using the comparative threshold (delta CT) method. Three independent biological replicates were performed for each experimental variant.

### 2.6. Computational Molecular Docking to Study the Interaction of ABA with LTPs

Interaction of ABA with pea, dill (*Anethum graveolens*), and lentil (*Lens culinaris*) LTPs was studied in silico. Solution structures of Ps-LTP1 [PDB ID: 2N81], Ag-LTP [2N2Z] and Lc-LTP2 [2MAL], determined by us earlier [3,19,22] using nuclear magnetic resonance methodology, were used to prepare protein structures for rigid receptor docking. 3D conformer of ABA was obtained from the PubChem database [PubChem CID: 5280896]. Preparation of each protein for docking, including removal of water molecules, protonation, correction of errors and missing structure data, and assignment of partial charges using the AMBER ff14SB force field, was carried out using the DockPrep tool of the UCSF Chimera v.1.4 software package (San Francisco, CA, USA) [23]. The docking box was chosen so that whole protein molecules in the ribbon representation were entirely within this box. Blind docking of ABA into hydrophobic cavities of plant LTPs, based on the Lamarckian genetic algorithm (LGA), was carried out using the AutoDock Vina tool of the UCSF Chimera v.1.4 software [24]. Interactions between the proteins and ABA were analyzed and visualized with the Discovery Studio Visualizer v20.1.0.19295 software [25].

### 2.7. Fluorescence Spectroscopy to Study of LTP Binding of ABA In Vitro

The ability of pea Ps-LTP1, dill Ag-LTP and lentil Lc-LTP2 (obtained as previously described [3,22]) to bind ABA was assayed using the fluorescent probe TNS (2-p-toluidinylnaphthalene-6-sulphonate). Lipid binding analysis was performed at 25 °C using an F-2710 spectrophotometer (Hitachi High Technologies America Inc., Pleasanton, CA, USA). The excitation and emission wavelengths were set at 320 and 437 nm, respectively. Before the initial fluorescence (F_0_) was recorded, 3 μM TNS with or without 10 μM of ABA was incubated for 1 min in 2 mL of a measurement buffer (175 mM mannitol, 0.5 mM K_2_SO_4_, 0.5 mM CaCl_2_, and 5 mM MES at pH 7.0) and then solution of the LTP (4 μM final concentration) was added. The equilibrated fluorescence (F) was recorded after 2-min incubating each of LTPs with TNS in mixture with ABA. The results were expressed as a percentage of the LTP–TNS complex with fluorescence calculated according to the formula [(F − F_0_)/F_C_] × 100%, where F_C_ is the fluorescence of the LTP–TNS complex in the absence of ABA.

### 2.8. Statistics

Data were expressed as means ± SE, which were calculated in all treatments using MS Excel. Significant differences between means were analyzed by t-test.

## 3. Results

To find out when the treatments resulted in physiological responses we measured transpiration. This had the advantage of being straightforward and likely to be related to suberin deposition, which we believe is linked to ABA and salt effects. Both ABA and salt treatment reduced transpiration (Table 1). A ≅4-fold decline in transpiration was noticeable already on the second day after the start of ABA-treatment, while the effect of salt-treatment was less marked, showing a 1.5-fold decline in transpiration, detectable only on the fourth day. In accordance with this, hydraulic conductance and other characteristics were measured on the second day in the case of ABA treatments and on the fourth day in the case of salt-treatment. Measurement of both transpiration and leaf water potential enabled the hydraulic conductance of the whole plant to be estimated. This was decreased approximately 6-fold and 3-fold by ABA and salt treatment respectively.

**Figure 1:**
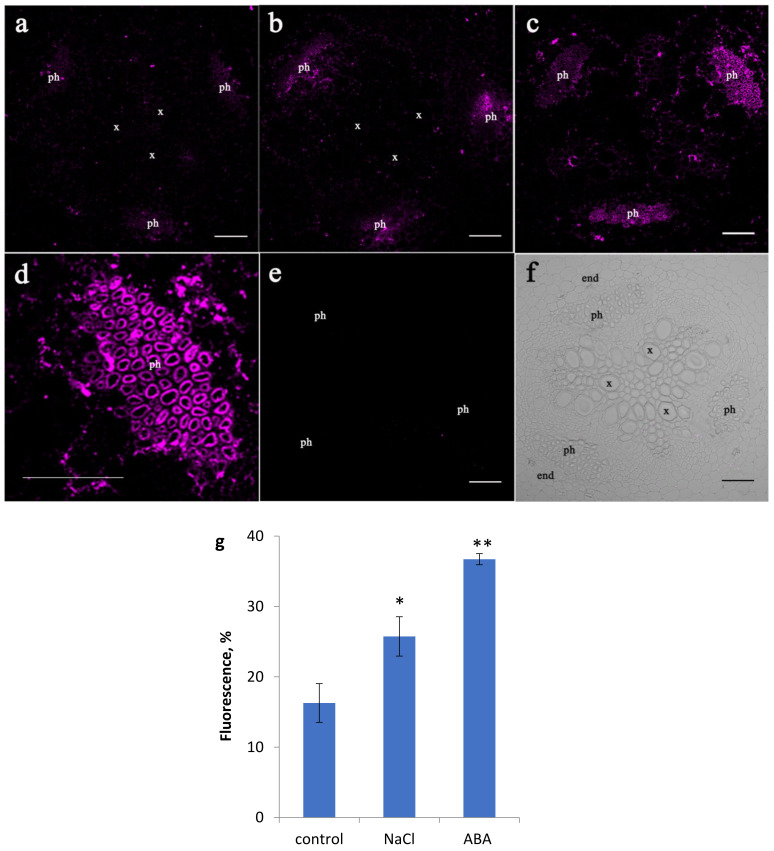
Effects of NaCl and ABA on LTP localization in the central cylinder of root basal part. Root section of pea plants grown without NaCl and exogenous ABA (**a**), physiological control), treated with NaCl (**b**) or ABA (**c**). Higher magnification image of the phloem cells from figure C (**d**). Immunohistochemical control with non-immune serum (**e**). The bright field image of the root central cylinder (**f**). Fluorescence for LTP in phloem (means ± SE, arbitrary units, maximal fluorescence taken as 100 %, minimal as 0 %) of control, NaCl and ABA-treated plants) (**g**). Means (n = 8) significantly different from control at *p* ≤ 0.05 and 0.001 (t-test) are marked with * and **, respectively. x – xylem; ph – phloem; end – endodermis. Scale bar is 100 µM.

An immunohistochemical study of sections of the roots of control pea plants (without treatment) using serum, containing polyclonal anti-LTP antibodies, showed weak fluorescence suggesting low abundance of LTPs (Figure 1a). Both NaCl and ABA treatments increased fluorescence brightness, especially in the phloem region within the central cylinder (Figure 1b,c,g). Higher magnification image of the phloem cells showed that LTPs was mainly present around the perimeter of the cells presumably in the cell walls (Figure 1d). The specificity of the method was confirmed by the absence of fluorescence in control sections processed with non-immune serum (Figure 1e).

**Figure 2:**
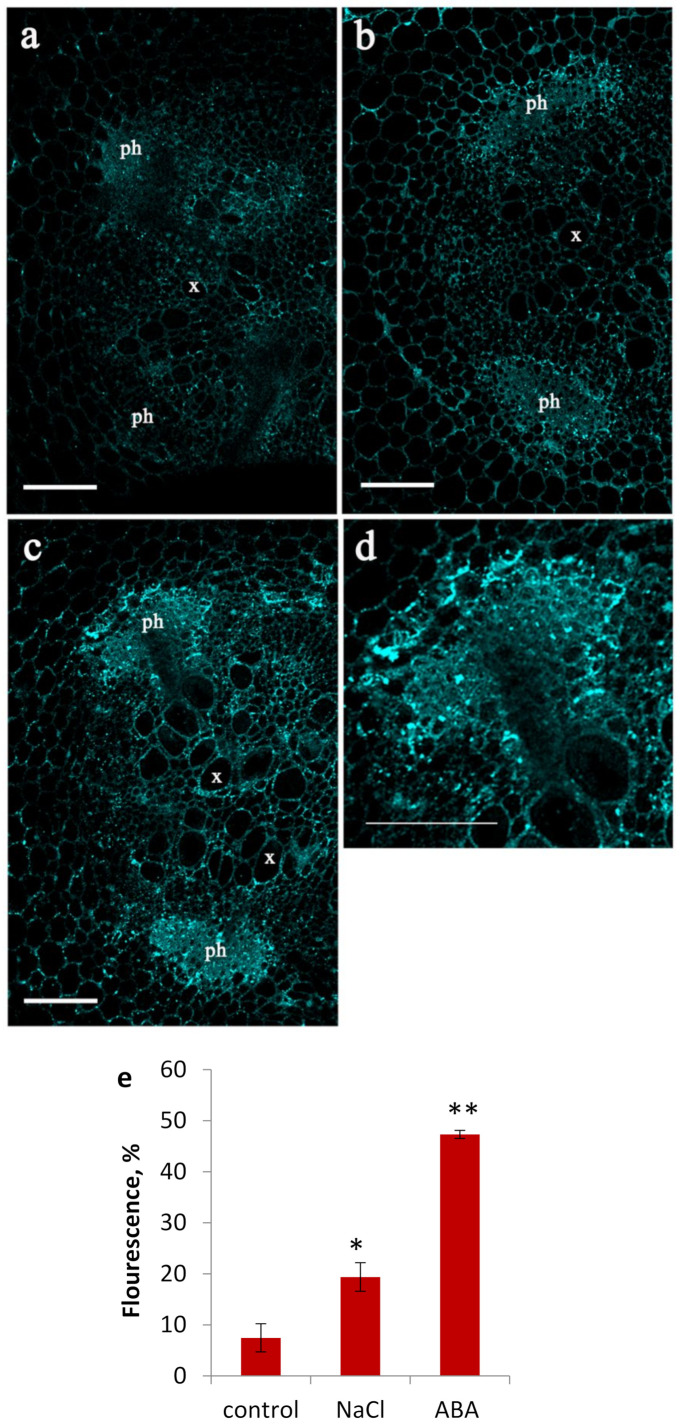
Effects of NaCl and ABA on ABA localization in the central cylinder of root basal part. Root section of pea plants grown without NaCl and exogenous ABA ((**a**), physiological control), treated with NaCl (**b**) or ABA (**c**). Higher magnification image of the phloem cells from figure C (**d**). Fluorescence for ABA in phloem (means ± SE, arbitrary units, maximal fluorescence taken as 100 %, minimal as 0 %) of control, NaCl and ABA-treated plants) (**e**). Means (n = 8) significantly different from control at *p*≤0.01 and 0.00001 (t-test) are marked with * and **, respectively. x – xylem; ph – phloem. Scale bar is 100 µM.

Both NaCl and ABA treatments resulted in a rise in fluorescence from immunolocalization of ABA using polyclonal rabbit anti-ABA sera (Figure 2), indicating increased ABA content in the roots of salt-treated plants. As in the case of LTPs, ABA-linked fluorescence mainly increased in the phloem region. However, unlike LTPs fluorescent signal of ABA was present not only around the perimeter of the cells but was also present within cells (Figure 2d).

**Figure 3:**
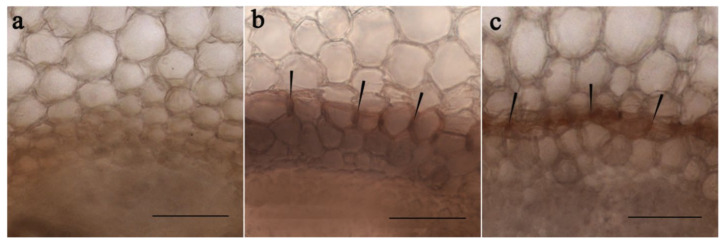
Effects of NaCl and ABA on suberin deposition. Root cross-sections in the endoderm region of pea plants grown under control conditions (**a**), treated with NaCl (**b**) or ABA (**c**). Suberin staining in the endodermis is indicated with arrows. Scale bar is 50 µM.

To check the involvement of salinity and ABA in deposition of suberin, histochemical staining for suberin using Sudan III was performed and revealed the suberin lamellae in the endoderm region of salt- and ABA-treated pea plants (Figure 3b,c). These suberin lamellae were not visible in root sections of control plants (Figure 3a).

**Figure 4:**
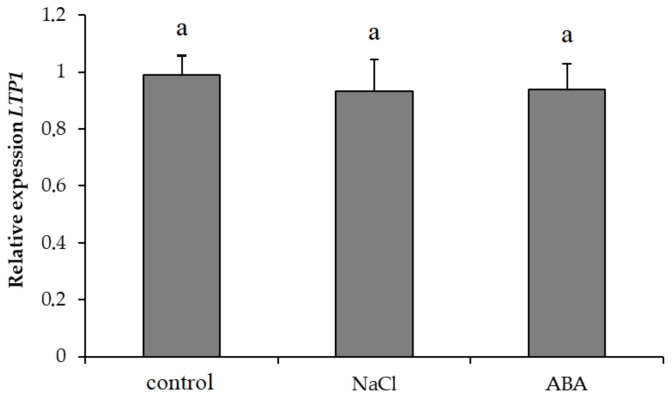
Abundance of transcripts of *Ps*-*LTP1* gene in pea roots treated with NaCl and ABA. Means ± SE are presented. Means for the control, NaCl and ABA treatment are marked with similar letters, which means that they were not significantly different (n = 3, t-test).

Neither salt nor ABA treatment influenced the abundance of *Ps*-*LTP1* gene transcripts (Figure 4).

**Figure 5:**
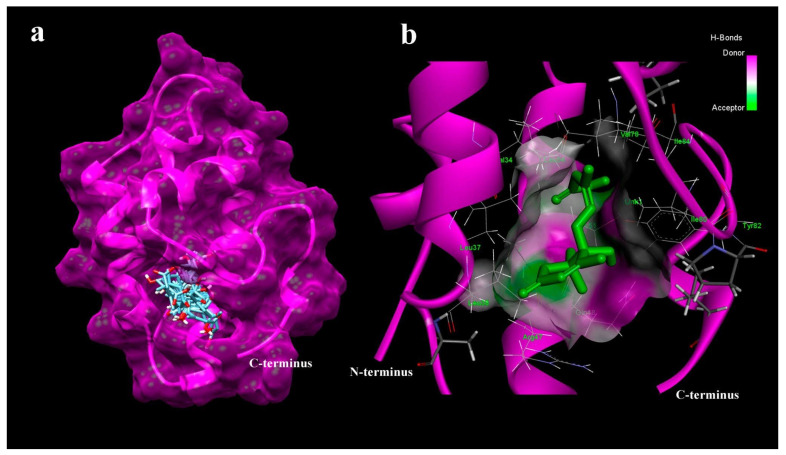
Spatial structure of pea Ps-LTP1 complexed with 10 conformations of ABA calculated by means of blind molecular docking (**a**). Interaction of Ps-LTP1 with best conformation of ABA found by AutoDock Vina (**b**).

It is well known that plant LTPs bind different hydrophobic ligands, including the plant hormone jasmonic acid and may thus take part in hormone signal transduction [15]. However, nothing is known about the ability of these proteins to bind ABA and the probability of them participating in its trafficking. Since one of the goals of the present research was to test the ability of LTPs to bind ABA, we investigated the binding of ABA with various LTPs from different plant species. Initially, the binding of pea Ps-LTP1, lentil Lc-LTP2 and dill Ag-LTP with ABA was investigated by means of blind molecular docking. AutoDock Vina found 10 conformations of abscisic acid (ABA) in the hydrophobic cavity of Ps-LTP1 with affinity energy ranging between −6.9 and −6.0 kcal mol^−1^ (Figure 5a). All 10 conformations of ABA were located close to the more hydrophilic “bottom” entrance of the cavity near the C-terminus. The following amino acid residues take part in the interaction of Ps-LTP1 with the best conformation of ABA according to the molecular docking calculations: Val34, Leu37, Leu38, Arg47, Gln48, Ala50, Cys51, Leu54, Val78, Ile80, Tyr82, and Ile84 (Figure 5b). For dill Ag-LTP, AutoDock Vina found 10 conformations of the ABA, fully immersed into the hydrophobic cavity, with affinity energy ranging between −7.5 and −7.0 kcal mol^−1^. Lentil Lc-LTP2, had the lowest hydrophobic cavity volume with AutoDock Vina finding 10 conformations of ABA outside the hydrophobic cavity with affinity energy ranging between −5.4 and −4.8 kcal mol^−1^.

**Figure 6:**
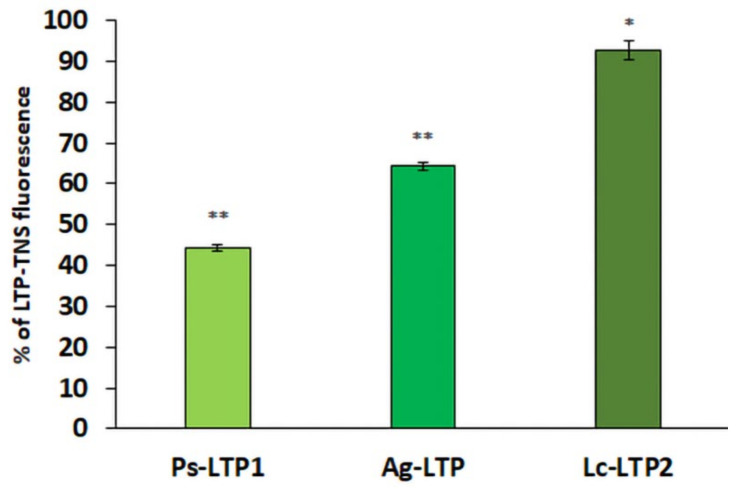
Effects of ABA on the fluorescence level of the LTP-TNS complexes. Ps-LTP1, Lc-LTP2 and Ag-LTP – LTPs from pea, lentil and dill. Results are expressed as the percentage of fluorescence of the LTP–TNS complexes. Means ± SE (n = 3) are presented. Means significantly different from control at *p* ≤ 0.05 and 0.001 (t-test) are marked with * and **, respectively.

To confirm the ability of plant LTPs bind to ABA in aqueous solution, TNS displacement assays were performed (Figure 6). TNS is one of a class of compounds, which do not fluoresce in water but fluoresce strongly in hydrophobic environments. Binding of TNS to the LTP hydrophobic cavities resulted in an increase in fluorescence intensity, the value of which was taken as 100% for each protein [26,27]. When the LTP was added to a mixture of TNS and ABA, the fluorescence of TNS became lower than that of the control when LTP was given to TNS alone. This permitted us to assess and compare affinities of ABA to the different LTPs (Figure 6). ABA was easily accommodated in the cavity of Ps-LTP1, reducing the fluorescence by 66%. For dill Ag-LTP, the protein-TNS fluorescence was reduced by 36% while the decrease in Lc-LTP2-TNS fluorescence was only 8%, indicating minimal ABA binding to this protein. Apparently, the observed increase in displacement of TNS from Lc-LTP2 to Ps-LTP1 is due to a greater hydrophobic cavity (Ps-LTP1 ~1000 Å^3^, Ag-LTP ~800 Å^3^, Lc-LTP2 ∼600 Å^3^).

## 4. Discussion

To test the hypothesis that ABA is involved in the control of LTP abundance in salt-stressed plants we compared the effects of NaCl and ABA on LTP accumulation in pea roots. Immunohistochemical localization showed that salinity increased pea LTP abundance in the central cylinder of basal root (Figure 1) accompanied by an increased content of ABA (Figure 2). ABA as well as LTP accumulation in salt-stressed plants was shown earlier [28,29]. But their co-localization in root tissues under salt stress was reveled in the present study for the first time. Not only salinity, but also ABA-treatment increased the abundance of LTPs in the same regions of pea roots. Based on our experimental results and literature concerning ABA-induced expression of LTP genes plants [13], an involvement of ABA in LTP accumulation in the roots of salt-stressed pea plants was indicated.

In earlier studies, Ps-LTP1, binding various lipid ligands had been purified from pea seeds and the *Ps*-*LTP1* gene shown to exhibit high expression in seedlings, leaves and tendrils of mature plants. *Ps*-*LTP1* transcripts were found also in pea roots [19]. Consequently, in the present work we investigated expression levels of *Ps*-*LTP1* under both salinity and ABA-treatment. However, we failed to detect effects of either NaCl or ABA on the abundance of Ps-LTP1 transcripts (Figure 4). It is well known that LTPs in plant genome are represented by a set of genes encoding different isoforms, which may perform various functions. We may thus have missed monitoring the most appropriate LTP transcript since there are many LTP isoforms in plants. Perhaps intensive immune staining of LTPs in salt-stressed and ABA-treated plants was associated with an increase in the level of expression of other LTP isoforms which could interact with polyclonal rabbit antibodies raised against LTP.

Finding a strong fluorescent ABA signal in the phloem of ABA-treated plants is an intriguing result (Figure 2c). It is of interest to explain how the applied hormone seemingly accumulated in this region. One explanation may be via recirculation through phloem of ABA initially transported to the shoot from the roots through xylem [30]. ABA may also accumulate in the phloem via trapping of this weak acid in alkaline compartments [12,31].

Increased abundance of LTPs in salt-stressed and ABA-treated pea plants coincided with deposition of suberin lamellae in the endodermis (Figure 3) thereby contributing to formation of a diffusion barrier preventing penetration of toxic Na^+^ ions into the stele of the root and further flow up to the shoots. These results are in accordance with hypothesized function of LTPs in cell wall suberization [1] and supported by the localization of LTPs around the perimeter of the phloem cells (presumably in the cell walls) located near the endodermis (Figure 1d). Suberin assembly in plant cell walls requires translocation of lipid compounds across the plasma membrane and passage of hydrophobic precursors through the hydrophilic cell wall for deposition of suberin polymer. LTPs are believed to be important for this process [32] although the roles of LTPs in suberin synthesis remain speculative [33]. The results obtained in the present research showing coincidence of increased abundance of LTPs (Figure 1) with increased deposition of suberin in the roots of ABA- and NaCl-treated plants (Figure 3) serve to support this hypothesis.

Measurement of hydraulic conductance of pea plants showed that it was decreased by salt treatment (Table 1), coinciding with formation of an apoplast barrier at the endodermis (Figure 3). Deposition of suberin is known to decrease hydraulic conductance resulting in reduced transpiration flow [34]. This is believed to enhance salt tolerance, by diminishing the flow of toxic ions within the transpiration stream [10]. Overexpression of LTP genes was shown as linked to reduced transpiration flow [9] resulting in increased stress tolerance by lessening the probability of leaf dehydration. This appears to be the first published evidence supporting the involvement of LTPs in the control of suberin deposition and hydraulic conductance in salt-stressed plants.

A strong fluorescent LTP signal was detected in the phloem cells of both salt- and ABA-treated pea plants (Figure 1). These results are in accordance with a report of GUS expression driven by the LTP promoter in root vascular bundles [32]. Phloem plays a crucial role as a major trafficking pathway for assimilates and other nutrients. However, until recently, lipid transport in the phloem has been given little attention [35], since transport of hydrophobic substances was not expected in the aqueous environment of sieve elements. However, a variety of lipids bound to proteins is found in human blood and transported to other tissues. This raises the possibility that lipids and their respective lipid-binding proteins in the phloem may serve similar functions in plants. This is supported by several reports of the presence of LTP and lipids in phloem [15,36].

In our case, pea LTP fluorescence was observed not throughout the phloem cells but mostly in cell walls (Figure 1). At the same time, the fluorescence of ABA, which was probably in the phloem sap, was distributed throughout the phloem cells (Figure 2). Therefore, we suggested the involvement of plant LTPs in phloem unloading of different lipids and signal lipid molecules. It is known that LTPs bind jasmonic acid and its associated complex. This is significant because this plant hormone is involved in triggering plant defense reactions [37]. Here we investigated the ability of LTPs to bind ABA using proteins of this class from pea, dill, and lentil and found these proteins also form complexes with ABA and that the binding efficiency depends on the structure and volume of their hydrophobic cavity (Figure 6). Pea Ps-LTP1 have the largest hydrophobic cavity among plant LTPs with an established spatial structure that showed the highest affinity to ABA compared to those from lentil and dill.

We speculate that this function of pea LTPs is particularly important under stress conditions such as salinity and ABA treatment since they both increase LTP accumulation in the root phloem. In particular, simultaneous increase of LTP accumulation in phloem cells and suberin deposition in pea root endodermis may indicate the possible participation of these proteins in the unloading of unsaturated fatty acids, which are the precursors of suberin. On the other hand, the simultaneous accumulation of LTPs and ABA in phloem cells may indicate the participation of these proteins in delivery of this plant hormone, which regulates the transfer of assimilates from the phloem to neighboring cells. The possibilities that plant LTPs may bind ABA and take part in ABA-mediated signal transduction will be the subject of further study.

## Figures and Tables

**Table 1:** Effect of ABA and salt treatment on transpiration (g plant^−1^ h^−1^), leaf water potential (LWP) and osmotic potential of nutrient solution (OPNS) (MPa) and hydraulic conductance (g plant^−1^ h^−1^ MPa^−1^) of pea plants. Means ± SE (n = 10) are presented. Means significantly different from control are marked with asterisk (*p* ≤ 0.05, t-test).

	Transpiration	LWP	OPNS	Hydraulic Conductance
	**2 days after ABA treatment**			
Control (without ABA)	0.23 ± 0.04	−0.4 ± 0.05	−0.01 ± 0.01	1.49 ± 0.24
ABA treatment	0.05 ± 0.01 *	−0.52 ± 0.04 *	−0.01 ± 0.01	0.27 ± 0.16 *
	**4 days after salt treatment**			
Control (without NaCl)	0.3 ± 0.02	−0.33 ± 0.08	−0.01 ± 0.01	0.93 ± 0.11
NaCl treatment	0.23 ± 0.03 *	−0.91 ± 0.1 *	−0.26 ± 0.04 *	0.35 ± 0.13 *

* *p* ≤ 0.05.
